# A novel open-type biosensor for the *in-situ* monitoring of biochemical oxygen demand in an aerobic environment

**DOI:** 10.1038/srep38552

**Published:** 2016-12-05

**Authors:** Takahiro Yamashita, Natsuki Ookawa, Mitsuyoshi Ishida, Hiroyuki Kanamori, Harumi Sasaki, Yuichi Katayose, Hiroshi Yokoyama

**Affiliations:** 1 Division of Animal Environment and Waste Management Research, Institute of Livestock and Grassland Science, National Agriculture and Food Research Organization (NARO), 2 Ikenodai, Tsukuba 305-0901, Japan; 2 Animal Husbandry Research Institute of the Kumamoto Prefectural Agricultural Research Center, 3801 Sakae, Koshi 861-1113, Japan; 3 Advanced Genomics Breeding Section, Institute of Crop Science, NARO, 1-2 Owashi, Tsukuba 305-8634, Japan

## Abstract

Biochemical oxygen demand (BOD) is a widely used index of water-quality assessment. Since bioelectrochemical BOD biosensors require anaerobic conditions for anodic reactions, they are not directly used in aerobic environments such as aeration tanks. Normally, the BOD biosensors are closed-type, where the anode is packed inside a closed chamber to avoid exposure to oxygen. In this study, a novel bioelectrochemical open-type biosensor was designed for *in-situ* monitoring of BOD during intermittent aeration. The open-type anode, without any protection against exposure to oxygen, was directly inserted into an intermittently aerated tank filled with livestock wastewater. Anodic potential was controlled using a potentiostat. Interestingly, this novel biosensor generated similar levels of current under both aerating and non-aerating conditions, and showed a logarithmic correlation (R^2^ > 0.9) of current with BOD concentrations up to 250 mg/L. Suspended solids in the wastewater attached to and covered the whole anode, presumably leading to the production of anaerobic conditions inside the covered anode via biological oxygen removal. Exoelectrogenic anaerobes (*Geobacter* spp.) were detected inside the covered anode using the 16S-rRNA gene. This biosensor will have various practical applications, such as the automatic control of aeration intensity and the *in-situ* monitoring of natural water environments.

Biochemical oxygen demand (BOD) is a measure of easily biodegradable organic compounds, and is one of the most widely used criteria for the assessment of water quality^1^. The standard method of BOD measurement is lengthy (typically 5 days: BOD_5_), and requires a trained technician to obtain reproducible results. Thus, the method is not suitable for process control in wastewater treatment and the real-time monitoring of water environments, such as rivers, streams, ponds, and ground water. The development of *in-situ* and real-time BOD monitoring techniques that can be directly applied to aerobic environments would advance current wastewater treatment methods, as well as aid in the preservation of water resources. Most notably, the proper automatic control of aeration duration, based on real-time BOD values, is desirable for the intermittent aeration process to efficiently remove both BOD and nitrogen from wastewater^2^. Many BOD biosensors designed to shorten the requisite measurement time have been developed^3^, including those based on bacterial respiration^4^,^5^,^6^, oxygen consumption by immobilised bacteria^7^,^8^, enzymatic reactions in dead cells^9^, and bioluminescence^10^. These biosensors measure BOD in short time—approximately 10 min to 1 hour—and show a high correlation with BOD_5_. However, these methods are complicated^3^, and the measurements are conducted *ex situ* (not *in situ*). Additionally, other sensors that use non-biological mechanisms have been reported for estimating BOD_5_, including those based on UV absorbance^11^ and fluorescence^12^. While these sensors conduct precise *in situ* monitoring of organic matters, the major drawback is that the sensors measure the total concentration of organic carbon, including non-biodegradable substances. In addition, these sensors are not suited for monitoring coloured wastewater and wastewater containing a high quantity of suspended solids (SS) owing to low light permeability^13^.

Bioelectrochemical systems (BESs) are emerging as an intriguing technology platform with various applications^14^. BESs comprise microbial fuel cells (MFCs) for the generation of electricity^15^ and microbial electrolysis cells (MECs) for the production of H_2_^16^. *Geobacter* spp. are representative exoelectrogenic bacteria and are frequently observed in the biofilm that develops on the anodes of BESs^17^. Originally, *Geobacter* spp. were isolated as Fe(III)-oxide reducing bacteria, which oxidise acetate to CO_2_, coupled with Fe(III) reduction, under anaerobic conditions^18^. These bacteria attach to the anodes of BESs, and transfer electrons to the anode via the oxidation of acetate^19^. Since the electron-transfer reaction also occurs only under anaerobic conditions, microbial BES-based devices require anaerobic environments in the vicinity of the anodes for current production. Thus, BESs-based biodevices are not currently directly applied in aerobic environments, such as on the interior of aeration tanks, and are usually designed as closed-type sensors, where the anode is packed inside a closed chamber to protect it from exposure to oxygen.

A BOD biosensor, based on a MFC using a bacterial consortium, was reported by Kim *et al*. in ref. 20. This biosensor was a two-chambered mediator-less MFC, and showed a strong linear correlation of charge with BOD_5_ concentrations up to 206 mg/L (R^2^ = 0.99). Another two-chambered MFC-based biosensor was developed for the continuous monitoring of BOD_5_ concentrations up to 200 mg/L^21^. A biosensor based on an air-cathode, single-chambered MFC displayed a linear correlation (R^2^ = 0.93) with BOD_5_ concentrations up to 350 mg/L^22^. Additionally, a BES-based biosensor with voltage input was demonstrated to have a wide detection range from 32 to 1,280 mg/L with a linear correlation (R^2^ = 0.97) of charge with BOD_5_^23^. However, these biosensors were performing *ex situ* monitoring under anaerobic conditions. An interesting biosensor, a submersible MFC-based device equipped with a blower to continuously aerate the cathode chamber, was developed for the *in situ* monitoring of BOD_5_ in groundwater, and showed a linear correlation of current with BOD_5_ up to 250 mg/L^24^. Although this biosensor was operated under anaerobic conditions, current production under aerobic conditions was not demonstrated. At present, there is no *in situ* BOD biosensor for direct use in an aerobic environment. Therefore, in the present study, we designed a novel configuration of an *in situ*, BES-based, open-type BOD (iBOB) biosensor that is directly applied during an intermittent aeration process. Furthermore, the bacterial community structure of the biofilm that developed on the anode was characterised in detail via next-generation sequencing technology using the 16 S rRNA gene.

## Results

### Current generation in aerobic environment

**Figure 1:**
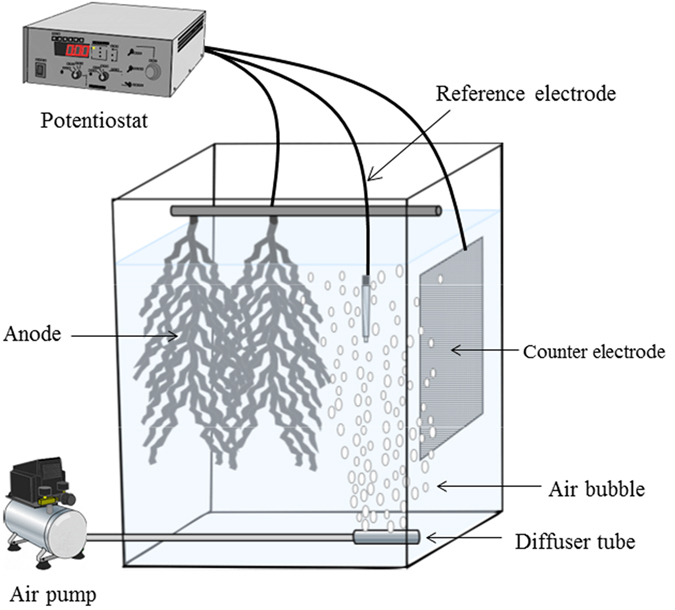
Schematic representation of the iBOB biosensor inserted into an intermittently aerating tank.

**Figure 2:**
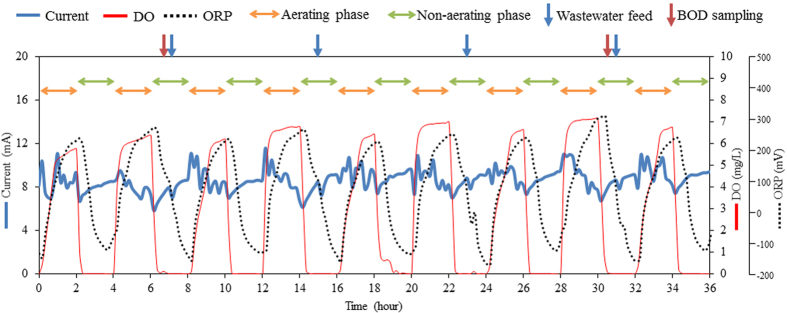
Current generation of the iBOB biosensor during aerating (orange arrows) and non-aerating (green arrows) phases . The aeration tank, fed periodically with the wastewater, was aerated in a cycle of 2 h aeration and 2 h non-aeration. The profiles of current, DO, and ORP are shown. The time points of wastewater feeding and sampling for BOD_5_ measurement are indicated with blue and red arrows, respectively.

**Figure 3:**
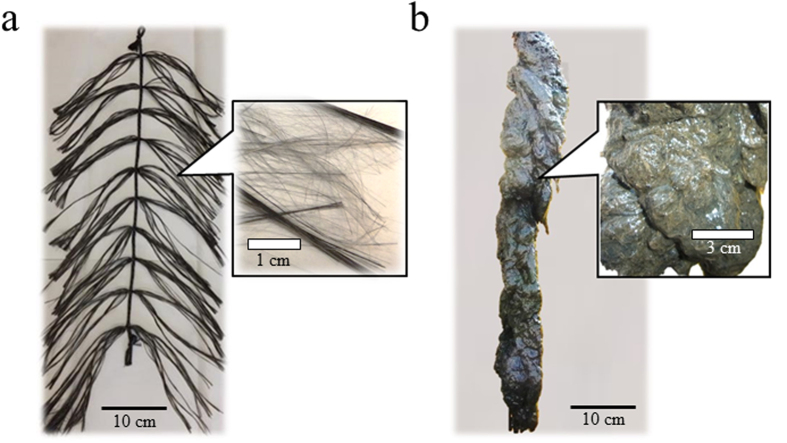
Anodes of the iBOB biosensor before (**a**) and after (**b**) use. The anode completely covered with SS, including hay feed, is shown.

The iBOB biosensor is a membrane-less device with an open-type anode (i.e. the anode is directly inserted into water without any protection against exposure to oxygen) (Fig. 1). The two anodes, along with an Ag/AgCl-reference electrode with a double junction and a counter electrode, were placed in a pilot-scale aeration tank (1.2 m^3^), and the anodic potential was controlled at a constant level using a potentiostat. The tank was intermittently aerated and was periodically supplied with livestock wastewater at a hydraulic retention time (HRT) of 4 days. To prevent adhesion of SS, the reference electrode was placed above a diffuser tube such that air bubbles from the tube directly struck the electrode and washed the SS away. In contrast, the anodes were not placed above the tube, ensuring tight adhesion of SS. After running for several weeks, pollutants (BOD_5_, SS, NH_4_-N and total nitrogen) in the wastewater were efficiently removed (>90%) by the intermittent aeration in a cycle of 2 h of aeration and 2 h of non-aeration (Table S1). The pH in the tank was in the range of 7.5–7.8. The dissolved oxygen (DO) of the wastewater was not detectable during the non-aerating phase, increasing in value to approximately 6–7 mg/L during the aerating phase (Fig. 2). The oxidation–reduction potential (ORP) of the wastewater also increased from −100 to −150 mV to 200–300 mV (vs. NHE) during aerating phase. The DO and ORP values conveyed that the aerobic and anaerobic environments repeatedly occurred in the tank as a result of the intermittent aeration. Bacteria naturally present in the wastewater functioned as the seed bacteria for current production. The iBOB biosensor stably generated current after approximately 2 to 3 weeks of operation. The current intensity in the non-aerating phase was 5.8–9.6 mA at an average of 8.2 ± 0.8 mA (Fig. 2). Interestingly, despite the aerobic environment, the biosensor also generated current during the aerating phase. The current intensity in the aerating phase was 6.9–11.6 mA, with an average of 9.0 ± 1.1 mA, which was slightly higher than the average observed in the non-aerating phase. This could be the result of the stirring of the wastewater by aeration, which may have been supplying fresh substrates and/or washing off inhibitory by-products from the anodic surface. SS, including small particles of hay feed, in the wastewater attached to and covered the whole surface of the anodes (Fig. 3). The amount of biomass attached was 29.4–37.9 g dry wt/g anode. Bacteria present on the outside area of the covered anodes were considered to consume oxygen, by which anaerobic environments could be produced inside the covered anodes. These results demonstrated that the iBOB biosensor is capable of generating current from the wastewater in the aerating phase.

### Monitoring of BOD_5_ in the intermittently aerating tank

**Figure 4:**
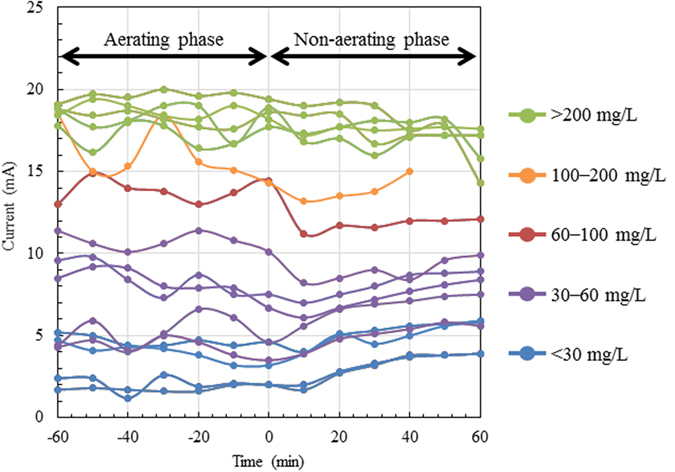
Profiles of current generation by the iBOB biosensor at various BOD_5_ concentrations in the intermittently aerated tank. The zero value on the horizontal axis indicates the time point of switching from the aerating to the non-aerating phase. BOD_5_ concentrations are indicated by colour.

**Figure 5:**
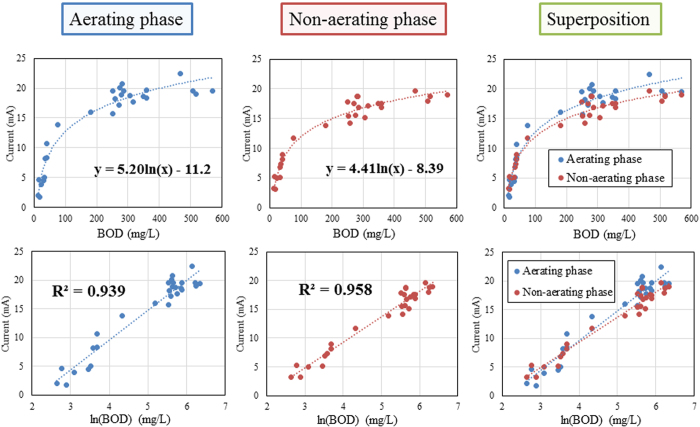
Correlation of current with BOD_5_ in aerating (left) and non-aerating phase (middle). Right panels are the superposition of left and middle panels. In the lower panels, the linear correlation of current with the values of logarithmically converted BOD_5_ is shown.

To analyse the relationship between current and BOD_5_ concentration, the tank was operated at different HRTs and aeration cycles, and BOD_5_ values ranging from 14 to 570 mg/L were obtained. The iBOB biosensor generated current at all BOD_5_ concentrations, and the levels and wave profiles of current generation appeared to be similar in both the aerating and non-aerating phases (Fig. 4). The current generation sharply increased with the increase in BOD_5_, ranging from 14 to 100 mg/L in both phases (Fig. 5, upper panels). Subsequently, the slope corresponding to current generation decreased with increasing BOD_5_ (>100 mg/L), and the current generation reached a plateau (17 mA) at 250 mg/L BOD_5_. The current displayed a logarithmic correlation with BOD_5_ in both phases (i.e. current was linearly correlated with the values of logarithmically converted BOD_5_) (Fig. 5, lower panels). The regression formulas for each phase are shown in Fig. 5, and the R^2^ values were greater than 0.9 in both phases. The superposed graphs in Fig. 5 (right panels) show that the generation of current in the aerating phase was nearly equal to or slightly higher than in non-aerating phase. On the basis of a t-test, the logarithmic coefficient in the regression formula for the aerating phase (5.20) was significantly higher (P < 0.05) than that for the non-aerating phase (4.41).

The biosensor stably generated current until the end of experiment (71 days), and no maintenance of the anodes, such as washing or replacement, was required. The amount of SS attached to the reference electrode was very slight, and the filter at the junction of the electrode appeared clean; no biofilm formation was observed on the surface. Deterioration of the counter electrode was not observed.

### Bacterial community structure

**Figure 6:**
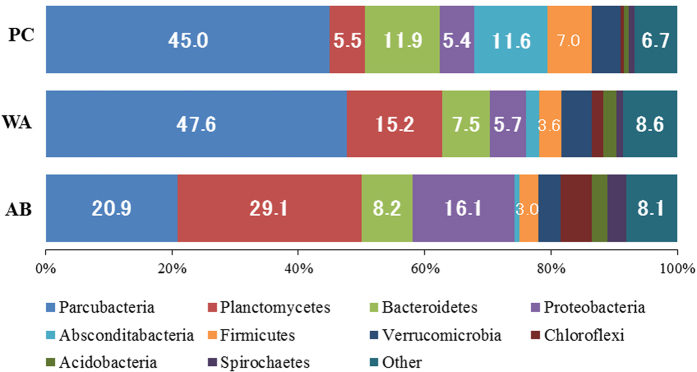
Phylum-level community structures of planktonic-cell (PC) fraction in the aeration tank and whole-anode (WA) and anode-binding (AB) fractions from the iBOB biosensor.

**Figure 7:**
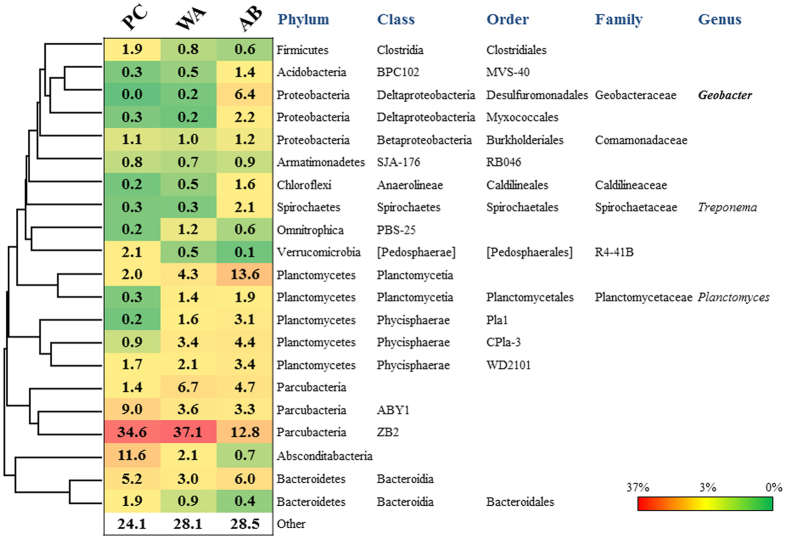
Phylogenetically clustered heat map of the major genera in the communities of the planktonic-cell (PC) fraction in the aeration tank and the whole-anode (WA) and anode-binding (AB) fractions from the iBOB biosensor.

To reveal the bacteria involved in the generation of current, the communities for the planktonic-cell fraction (PC) in the aeration tank and the whole-anode (WA) and anode-binding (AB) fractions from the iBOB biosensor, were analysed using high-throughput sequencing of the 16 S rRNA gene. The AB sample was prepared by washing the WA sample until the visible SS that were adhered to the anode disappeared. The distribution of the operational taxonomic units (OTUs) and alpha diversity of the communities are shown in Table S2. Based on the alpha-diversity indices, the level of diversity was similar among the communities. The rarefaction curve suggested that the number of OTUs observed did not reach a plateau in all communities (Fig. S1). Planctomycetes was the most abundant phylum (29%) in the AB sample, whereas the candidate phylum Parcubacteria^25^, previously called OD1, was the most predominant (45–48%) in the PC and WA samples (Fig. 6). The phylum Proteobacteria, which includes exoelectrogenic and metal-oxide reducing bacteria, was detected in the AB sample at a higher frequency (16%) than in the PC and WA samples. In the genus-level analysis (Fig. 7), the exoelectrogenic genus *Geobacter* in the AB sample was observed at a significantly higher frequency (6.4%) than that in the PC (0%) and WA (0.2%) samples. The class Planctomycetia, in the phylum Planctomycetes, in the AB sample occurred at a higher frequency (13.6%) than that in the PC and WA (2.0–4.3%) samples.

## Discussion

This study is the first report demonstrating the direct application of a novel biosensor, iBOB, in an aerobic environment for the *in situ* monitoring of BOD_5_. The most distinctive feature of the iBOB biosensor is the novel open-type configuration, where anodic potential is controlled at a constant level using a potentiostat, and the probe can be directly inserted into an intermittently aerated tank without any protection against exposure to oxygen. Thus far, BES-based biodevices, including MFCs, have been used in anaerobic environments, as anaerobic conditions are essential for the anodic reactions. Despite the aerobic environment, iBOB generated current during the aerating phase, likely via biological oxygen removal on the outer region of the anode covered with high amounts of SS. *Geobacter* spp., which are strictly anaerobic^18^, were observed in the iBOB biosensor, indicating that anaerobic conditions were produced inside the covered anode. *Geobacter* spp. could be mainly responsible for the generation of current in the iBOB biosensor via similar mechanisms observed with MFCs. In a preliminary experiment using a small-size anode, intermittently aerating tank (10 L), and artificial wastewater, we observed current production of the iBOB biosensor in the aerating phase, demonstrating the reproducibility of the BOD sensing under aerobic conditions (Fig. S2). Thus, this study suggests that, as a result of a thick biofilm on the anode, the application scope of BES-based biodevices is extended from anaerobic to aerobic environments. In addition, although the phylum Planctomycetes is rarely detected at a high frequency on MFCs, this phylum was detected at a high frequency on the iBOB biosensor. The class Planctomycetia in this phylum includes a diverse array of bacteria, such as aerobic heterotrophic bacteria and anaerobic ammonium oxidation (anammox) bacteria, which oxidise ammonia into N_2_ gas using nitrite as an oxidising agent under anaerobic conditions^26^. Despite this, there are no known exoelectrogenic members of the class Planctomycetia. The Planctomycetes-dominant community structure observed in our study is an intriguing signature of the iBOB biosensor, although the role of Planctomycetes in the generation of current is unclear.

MFC-based BOD biosensors frequently show linear correlations of current or charge with BOD_5_. However, in the present study, we observed a logarithmic correlation of current with BOD_5_ for the iBOB biosensor. In MFCs, current generation is dependent on both anode and cathode reactions, where the cathode reaction is known to have a substantial effect on current production. When the Pt catalyst of the cathode is poisoned, the MFC-based BOD sensor will decrease current production, leading to an error signal. On the other hand, the anodic potential is kept continuously constant using a potentiostat in the iBOB biosensor, and thus, the current generation is dependent only on anodic conditions. In this regard, the iBOB biosensor is predicted to generate current in a more stable manner than MFC-based biosensors. With this information in mind, it stands to reason that the regression formula for the iBOB biosensor might be different from those of MFC-based biosensors as a result of their dependency on the cathode. We observed that current generation of the iBOB biosensor sharply increased until BOD_5_ concentrations reached 100 mg/L in both the aerating and non-aerating phases, and subsequently reached a plateau at 250 mg/L. Therefore, we conclude that the range of high sensitivity detection for BOD_5_ in livestock wastewater is 14 to 100 mg/L, and that low sensitivity detection is possible up to 250 mg/L. Nearly identical, but not equal, regression formulas can be used in both phases of intermittent aeration.

iBOB does not require an ion-exchange membrane or protection of the anode against exposure to oxygen. The biosensor can be readily set up at existing aeration tanks and water environments. The biosensor displayed the accurate monitoring of BOD_5_ with R^2^ values (>0.9) in both the aerating and non-aerating phases. Moreover, the anode of the biosensor did not require any maintenance (e.g. washing), even though the anode was completely covered with SS. On the other hand, when a DO or ORP sensor is continuously used in aeration tanks, the sensor signal tends to be unstable because of membrane fouling with SS in the absence of automatic washing of the probe. In contrast, not only does the anode of iBOB require less maintenance, the adhesion of SS to the anode is beneficial in that it leads to an anaerobic environment on the interior of the attached anode. The adhesion of SS to the reference electrode was very limited because the air bubbles from the diffuser tube washed away the SS in the aerating phase. In pH and OPR probes, the key components (H^+^-absorbing Li-glass membrane and metal electrode, respectively) for the measurements are directly exposed to the solution measured; thus, impurities in wastewater are liable to adhere on the components. However, in the Ag/AgCl reference electrode of the present study, the key component for measurement is not exposed to the outer solution, and the inner solution is simply separated from the outer solution through a non-selective junction filter. In this study, the filter of the reference electrode appeared to be clean as a result of washing with air bubbles throughout the experiment, and no biofilm formation was observed on the surface. The inside of the reference electrode was filled with 3.3 M KCl, and the salt was capable of leaking from the filter very slowly. Biofilms would therefore not form on the surface of the filter because of the high salinity of KCl. In addition, the reference electrode used in this study was of the double-junction type, which has an extra barrier to protect the interior Ag. As compared to single-junction electrodes, double-junction electrodes are more resistant to poisoning of Ag by infiltration of impurities such as protein and sulfides, and thus have a longer lifetime. The use of a double-junction electrode would contribute to the accurate BOD sensing of iBOB.

The development of *in situ* and real-time BOD monitoring techniques that can be directly applied to aeration tanks will have a considerable impact on process control in wastewater treatment situations, especially for the automatic control of aeration intensity in small-scale treatment plants for on-site purification, such as is necessary at livestock farms and food processing factories. In summary, the iBOB biosensor has the following potential practical applications: (i) the discharge of inadequately treated wastewater with high BOD concentrations to surrounding water environments can be prevented using a signal from the biosensor. (ii) As aeration consumes considerable electrical power in wastewater treatments, the biosensor produces a stop signal when the BOD concentration of wastewater decreases to an effluent standard to avoid excess aeration and decrease associated costs. (iii) The importance of nitrogen removal from wastewater is currently increasing to protect water environments from further eutrophication^27^. The control of the ratio of BOD/nitrogen is critical in the nitrogen removal by the intermittent aeration process^2^. This is especially true for wastewater with a low BOD/nitrogen ratio^28^, such as in faeces/urine-separated livestock wastewater and the effluence from biogas plants, as excess aeration depletes BOD as an electron donor, which is required for biological denitrification. Using a signal from iBOB, the ratio of BOD/nitrogen could be properly maintained by controlling the aeration duration in the intermittent aeration process. (iv) The unique configuration of the open-type anode allows the setup of the iBOB to be easily tailored to various water environments, such as ponds and streams. In conclusion, this biosensor will be instrumental in monitoring the pollution of vital water resources. Further studies, such as the evaluation of regression formulas for various kinds of wastewaters and natural waters, are needed to fully document the practical applications of the iBOB biosensor.

## Conclusions

We designed a novel BES-based biosensor, equipped with an open-type anode, for the *in situ* monitoring of BOD_5_ under conditions of intermittent aeration. The iBOB biosensor generates current in both aerating and non-aerating phases of intermittent aeration, exhibiting a high correlation (R^2^ > 0.9) with BOD_5_. The biosensor can be easily set up in existing aeration tanks as well as in natural environments. The iBOB biosensor will be useful in myriad applications, most notably for the automatic control of aeration intensity.

## Methods

### Operation of the intermittent aeration reactor

The reactor was a cuboidal-shaped pilot-scale tank with an interior volume of 1.2 m^3^. The livestock wastewater, including manure and rinse water, was collected from the cattle and swine barns at Institute of Livestock and Grassland Science (Tsukuba-city, Ibaraki, Japan). Wastewater was sieved using a screw press, and the filtrate was periodically pumped to the tank at a flow rate of 33 L/min for 2 min every 8 h, corresponding to the HRT of 4 days. The water level was maintained at 800 L by overflow control, and the water temperature was kept at 22 °C by inserting two electrical heaters (500 kW) into the tank. The tank was intermittently aerated via diffuser tubes by using an XP30 air-pump (Techno Takatsuki, Osaka, Japan) at a flow rate of 3 m^3^/m^3^·h in a cycle of 1 h of aeration and 2 h of non-aeration for a week from the beginning of operation, and thereafter, the aeration cycle was changed to 2 h of aeration and 2 h of non-aeration. After running under this condition for several weeks, the reactor was operated at different HRTs and aeration cycles to obtain various concentrations of BOD_5_.

### Development of the iBOB biosensor

The anode (also called the working electrode) consisted of carbon-fibre bundles (26.7 g), and formed a tree-shaped structure with a stem (70 cm) and 20 branches (20 cm). Each branch contained 144,000 polyacrylonitrile-based carbon fibres with a diameter of 7 μm (Mitsubishi Rayon, Tokyo, Japan). Two anodes were hung in the aeration tank, and were connected to the potentiostat HAL-3001A (Hokuto Denko, Tokyo, Japan) using a copper wire. An Ag/AgCl-reference electrode of a double-junction type with porous ceramic filters (International Chemistry, Chiba, Japan) and a counter electrode made of stainless-steel mesh (60 mesh, SUS304, 90 cm × 80 cm × 0.1 cm in size) were also connected to the potentiostat. The anode potential was controlled at −0.2 V (vs. Ag/AgCl) with the potentiostat throughout the duration of the experiments. Current generation was recorded every 10 minutes with a data logger ZR-RX45 (OMRON, Kyoto, Japan). DO, pH, and ORP were monitored using the YUSB-01DO, YUSB-01PH, and YUSB-01OR indicators, respectively (DKK-TOA Yamagata, Yamagata, Japan).

### BOD_5_ measurement and chemical analysis

To determine BOD_5_, treated wastewater was sampled from the aeration tank once per a day. BOD_5_ was measured at 20 °C via a respirometric method by using a BODTrack^TM^ apparatus equipped with a pressure sensor (Hach, Düsseldorf, Germany), in the presence of a nitrification inhibitor for 5 days. Concentrations of NH_4_-N, NO_2_-N, and NO_3_-N were determined using an ion chromatograph, IC-2010 (Tosoh, Tokyo, Japan). Total nitrogen was analysed with a total carbon and nitrogen analyser, TOC-V CSN (Shimadzu, Kyoto, Japan).

### Analysis of community structure

High-throughput sequencing was performed via the MiSeq Illumina sequencing platform (Illumina Inc., CA, USA) using the region from V3 to V4 from the 16S rRNA gene^29^. A part of the carbon-fibre anode was cut off, and was roughly washed with distilled water. The bacteria present in the excised anode were regarded as the WA fraction. Subsequently, the excised anode was further washed with distilled water, until the visible SS attached to the anodic surface completely disappeared. The bacteria binding to the strongly washed anode were regarded as the AB fraction. The wastewater in the tank was sampled, and was centrifuged at 1,000 *g* for 10 min. The precipitate was washed with distilled water, and the bacteria in the precipitate were regarded as the PC fraction. Genomes were extracted from the fractions using an UltraClean™ Soil DNA Isolation kit (Mo Bio Laboratories, CA, USA). Libraries were constructed in a two-step PCR using the primers, including the Illumina overhang adapter sequences, according to the manufacturer’s instructions. The libraries were sequenced on 300PE MiSeq run, and the read sequences were clustered into OTUs using the Uclust method^30^ at a similarity threshold of 97% using QIIME software^31^. Representative sequences were aligned using PyNAST^32^, and the taxonomic classification and alpha diversity indices were computed with QIIME. The phylogenetic tree, combined with the heat map, was calculated by the unweighted pair-group method using arithmetic averages (UPGMA) using MEGA4^33^.

## Additional Information

**How to cite this article**: Yamashita, T. *et al*. A novel open-type biosensor for the *in-situ* monitoring of biochemical oxygen demand in an aerobic environment. *Sci. Rep.* **6**, 38552; doi: 10.1038/srep38552 (2016).

**Publisher's note:** Springer Nature remains neutral with regard to jurisdictional claims in published maps and institutional affiliations.

## Supplementary Material

Supplementary Information
