# Supplementary material for: A novel open-type biosensor for the in-situ monitoring of biochemical oxygen demand in an aerobic environment
Source: Sci Rep. 2016 Dec 5;6:38552. doi: 10.1038/srep38552 (PMC5137150; doi:10.1038/srep38552)
Supplement: Supplementary Information [file srep38552-s1.pdf]

Supplementary Information

A novel open-type biosensor for the in-situ monitoring of biochemical oxygen demand in an aerobic environment.

Takahiro Yamashita, Natsuki Ookawa, Mitsuyoshi Ishida, Hiroyuki Kanamori, Harumi Sasaki, Yuichi Katayose, and Hiroshi Yokoyama.

Table S1. Performance of the intermittently aerated tank in terms of pollutant removal.

	BOD (mg/L)	SS (mg/L)	NH_4-N (mg/L)	NO_2-N (mg/L)	NO_3-N (mg/L)	Total nitrogen (mg/L)
Influent	1,212 ± 154	2,702 ± 357	78 ± 6.0	n.d.	n.d.	140 ± 13.7
Effluent	28 ± 3.5	32 ± 3.8	n.d.	0.1 ± 0	4.4 ± 0.9	13 ± 1.0
Removal percentage	97.7 %	98.8 %	100 %	-	-	90.7 %

The tank was supplied with livestock wastewater at an HRT of 4 days and was aerated in a cycle of 2 h aeration and 2 h non-aeration. n.d.: not detected.

Table S2. Number of reads and alpha diversity analysis of communities for the planktonic-cell (PC) fraction in the aerated tank and the whole-anode (WA) and anode-binding (AB) fractions in the iBOB biosensor..

Sample	No. of reads	No. of OTUs	Chao1 richness	Abundance-based coverage estimator	Shannon's diversity index	Good's coverage
PC	73,367	2,147	2,239	2,269	7.714	0.9966
WA	41,822	2,335	2,507	2,562	8.532	0.9907
AB	43,613	2,176	2,297	2,315	9.030	0.9939

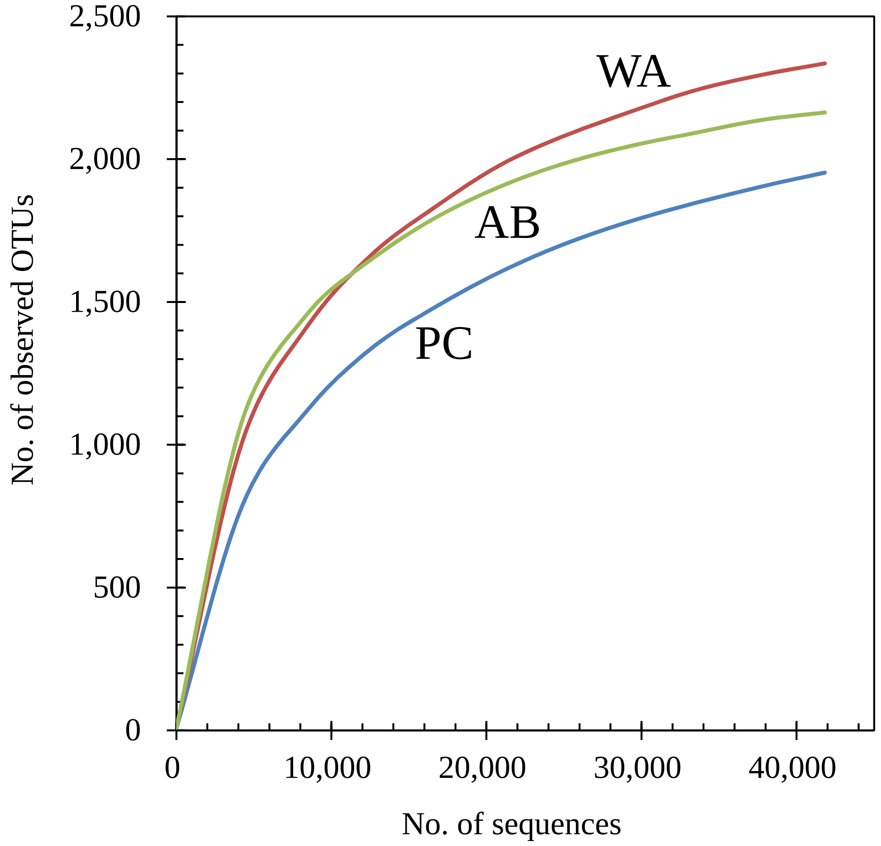

Figure S1. Rarefaction curves of read sequences for the communities of the planktonic-cell (PC) fraction (blue) in the aerated tank and the whole-anode (WA, red) and anode-binding (AB, green) fractions in the iBOB biosensor.

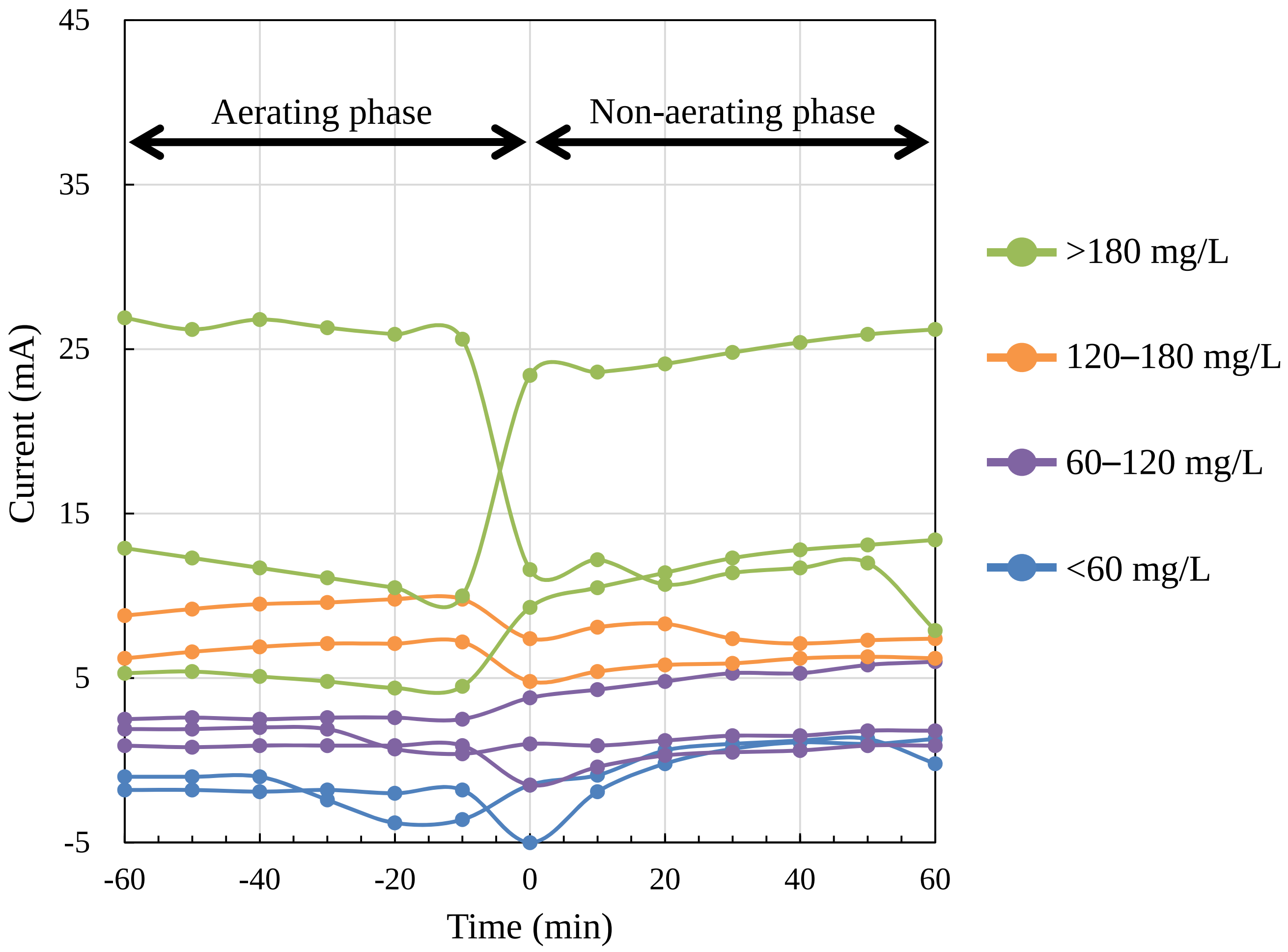

Figure S2. Result of the preliminary experiment using a small iBOD biosensor. The anode, the size of which was one sixth that of the anode shown in the main text, was inserted into a small aeration tank (10 L), fed with artificial wastewater containing 0.32 g/L potassium acetate and 0.22 g/L beef extract. The tank was intermittently aerated via diffuser tubes in a cycle of 2 h aeration and 2 h non-aeration. Profiles of current generation at various concentrations of chemical oxygen demand (COD) in the intermittently aerated tank are shown. The zero value on the horizontal axis indicates the time point of switching from the aerating to the non-aerating phase. COD concentrations are indicated by colour. Despite aeration, the biosensor generated a current in both the aerating and non-aerating phases, and the intensity of the current was positively correlated with COD.
